# Anthocyanin-Driven Dark Phenotypes in Stress Adaptation

**DOI:** 10.3390/plants15121870

**Published:** 2026-06-16

**Authors:** Chuzheng Zhang, Chenhao Wang, Zishan Ahmad, Yuxin Ye, Jinyi Cheng, Muthusamy Ramakrishnan, Qiang Wei

**Affiliations:** State Key Laboratory of Tree Genetics and Breeding, Co-Innovation Centre for Sustainable Forestry in Southern China, Bamboo Research Institute, Key Laboratory of National Forestry and Grassland Administration on Subtropical Forest Biodiversity Conservation, School of Life Sciences, Nanjing Forestry University, Nanjing 210037, China; m17372788520@163.com (C.Z.); wangchenhao0521@163.com (C.W.); ramky@njfu.edu.cn (M.R.)

**Keywords:** anthocyanin-driven dark phenotype (ADP), MBW regulatory complex, epigenetic regulation, plant stress adaptation

## Abstract

Anthocyanin-rich dark pigmentation is increasingly recognized as more than a simple consequence of flavonoid accumulation. Here, we define the anthocyanin-driven dark phenotype (ADP) as a coordinated stress-responsive state characterized by intense anthocyanin accumulation coupled with cellular and regulatory reprogramming. Recent studies show that reactive oxygen species, sugar signaling, temperature stress, and hormonal crosstalk converge on MYB–bHLH–WD40-centered regulatory networks that integrate pigment biosynthesis with vacuolar organization, transport activity, and stress adaptation. Epigenetic remodeling, chromatin dynamics, and post-transcriptional regulation further influence pigment intensity and persistence. Importantly, ADPs do not represent an alternative biosynthetic pathway or merely pigment abundance, but instead reflect a systems-level regulatory state governed by coordinated transcriptional, hormonal, and epigenetic control of the canonical anthocyanin machinery. However, several important questions remain unresolved, including how plants retain phenotypic stability under various environmental and developmental settings, whether ADPs contribute to long-term stress memory, and how anthocyanin accumulation is balanced with growth and energy expenditures. To translate ADP-associated features into crop development techniques, these gaps must be filled. We also emphasize spatial omics and CRISPR-based engineering as new methods for analyzing and modifying stress-resilient phenotypes.

## 1. Introduction

Plants often use color as an indicator of their internal physiological state. Anthocyanin accumulation is a common response to environmental stress and is often associated with tissues that appear deeply red-purple to nearly black. Traditionally, these darkened tissues have been interpreted as the quantitative outcome of enhanced flavonoid biosynthesis. However, accumulating evidence suggests that intense anthocyanin accumulation represents a coordinated stress-responsive state rather than pigment production alone [1]. High-intensity anthocyanin accumulation is regulated by MYB–bHLH–WD40 (MBW)-cantered networks that integrates oxidative bursts, sugar and energy signaling, temperature fluctuations, light responses, and hormonal crosstalk [2,3].

Anthocyanin-based pigmentation is also thought to be an important adaptive trait in the evolution of land plants, where increased photoprotection, antioxidant activity and environmental sensing probably helped to survive under changing terrestrial stresses. In this review, we define the anthocyanin-driven dark phenotype (ADP) as a stress-associated regulatory state in which high-density anthocyanin accumulation is coupled with cellular and physiological reprogramming. ADPs therefore reflect coordinated regulation of the canonical anthocyanin biosynthetic system rather than an alternative pigmentation pathway. ADP is therefore proposed as a systems-level interpretation of anthocyanin accumulation that integrates regulatory, metabolic, and physiological responses, rather than representing a distinct biosynthetic pathway or merely a quantitative increase in pigment content. Importantly, ADP should be distinguished from conventional anthocyanin accumulation phenotypes. Anthocyanin buildup is mainly explained in classical models as a quantitative metabolic reaction that alters color. On the other hand, ADP is a coordinated regulatory state that integrates metabolic, hormonal, transcriptional, and epigenetic layers to stabilize high anthocyanin output under stress. Therefore, not all anthocyanin-rich phenotypes are ADPs, even though all ADPs involve anthocyanin accumulation. This distinction is crucial because ADPs place more emphasis on stress-state integration and system-level regulation than just pigment intensity. According to recent research, pigment intensity, geographic distribution, and phenotypic stability are all influenced by chromatin accessibility, post-transcriptional regulation, vacuolar sequestration, and transporter function [1,4]. Anthocyanins are increasingly recognized as active modulators of stress physiology rather than passive stress-associated pigments. High anthocyanin accumulation can influence redox dynamics, vacuolar ion balance, and internal light environments, thereby contributing to photoprotection and cellular stabilization under stress [5,6,7]. The idea that multilevel regulatory networks govern ADP production is further supported by recent developments in single-cell transcriptomics, spatial metabolomics, transporter biology, and CRISPR-based regulatory interrogation.

Here, we review the physiological importance of anthocyanin-based dark phenotypes in stress adaptation, the regulatory mechanisms involved, and emerging engineering strategies for the development of stress-tolerant crops. We also highlight important open questions, including how plants maintain phenotypic stability across developmental stages and changing environments, whether persistent pigmentation contributes to stress memory, and how plants balance anthocyanin accumulation with growth and metabolic costs.

## 2. What Defines the Anthocyanin-Driven Dark Phenotype?

**Figure 1:**
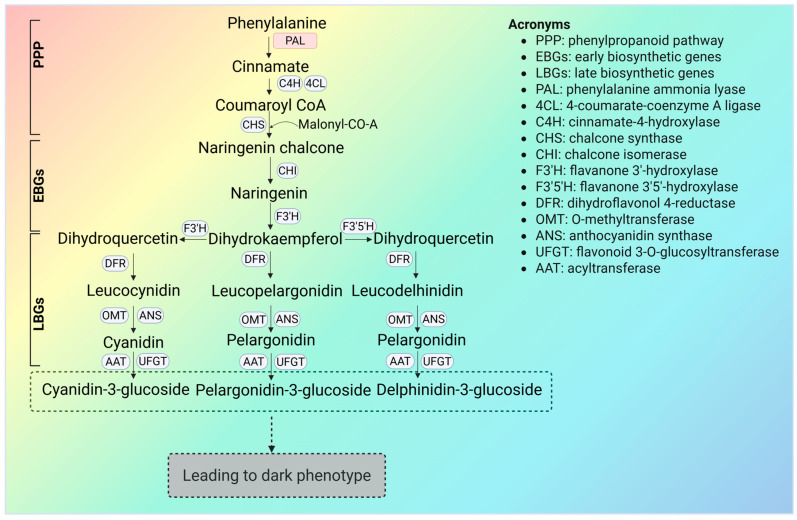
Anthocyanin biosynthesis in plants.

The anthocyanin-driven dark phenotype (ADP) should not be interpreted simply as increased pigmentation intensity, but as a coordinated, stress-associated regulatory state. The development of deep red-purple to nearly black tissues results from the integration of pigment production, vacuolar organization, cellular specialization, and stress-responsive regulatory networks, although increased anthocyanin accumulation is required for visible darkening (Figure 1). Recent research indicates that darkness arises from the coordinated enhancement of anthocyanin production along with physiological and structural processes that optimize optical density under stress [6,8]. Importantly, ADP differs from conventional anthocyanin-rich or purple/black pigmentation phenotypes, which primarily reflect quantitative increases in flavonoid content. In contrast, ADP represents a system-level regulatory state characterized by coordinated activation of stress-responsive networks rather than pigment accumulation alone. Thus, ADP should be distinguished from classical anthocyanin pigmentation, which is largely defined by metabolic output without necessarily involving integrated regulatory reprogramming.

At the biochemical level, multiple anthocyanin modifications contribute to the formation and stabilization of dark phenotypes. Anthocyanin acylation, glycosylation, and co-pigmentation significantly affect perceived darkness at the biochemical level by boosting pigment stability, red-shifted absorbance, and π-stacking interactions [9,10]. These modifications promote the formation of highly absorbing pigment states that increase tissue coloration and reduce reflectance. In addition to biochemical modifications, cellular and subcellular organization further intensifies tissue darkening. Optical absorbance at the cellular level is further increased by high-density pigment sequestration via phase-separated condensates and anthocyanic vacuolar inclusions (AVIs) [8]. Furthermore, specific epidermal or sub-epidermal pigment sink cells that improve light attenuation through multilayered optical organization are commonly seen in dark tissues, according to spatial-omics research [11]. Pigment accumulation and stability under stress circumstances are further influenced by vacuolar pH, ionic composition, and transporter activity, including GST-, MATE-, and ABC-mediated transport [12]. The establishment of ADP, therefore, requires coordinated regulation across biosynthetic, transport, storage, and stress-responsive pathways. Stress-associated signals like ROS bursts, sugar buildup, and hormonal cues such as ABA and JA, coordinately regulate MBW-complex activity, vacuolar transport systems, and co-pigment biosynthesis to optimize photoprotection, redox buffering and stress resilience. The dynamic induction and reversibility of the phenotypic are also influenced by post-translational and epigenetic control [13]. Together, these findings support the view that anthocyanin-driven dark phenotypes represent integrated stress-responsive regulatory configurations rather than simple quantitative extensions of anthocyanin accumulation.

## 3. Stress Signaling and Anthocyanin-Driven Dark Phenotypes

### 3.1. ROS Signaling as an Upstream Driver of ADP Formation

**Figure 2:**
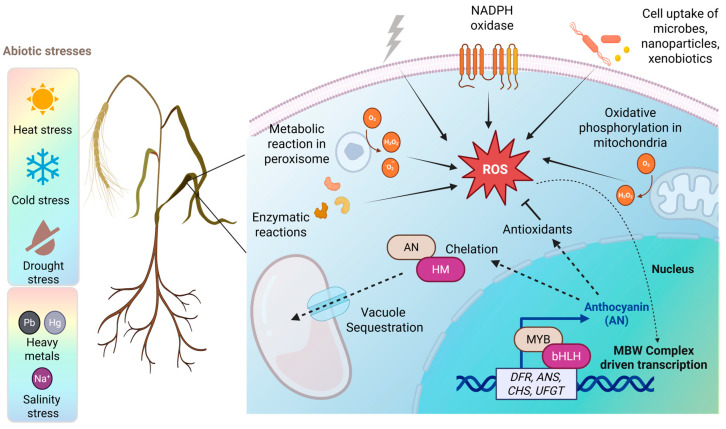
Protective roles of anthocyanins during stress-induced oxidative and metal toxicity. Abiotic stressors such as heat, cold, dehydration, salinity and heavy metal exposure induce intracellular ROS generation via metabolic events, enzymatic processes, NADPH oxidase activity and mitochondrial oxidative phosphorylation. Increased ROS levels trigger MYB–bHLH–WD40 (MBW) transcriptional complex that regulates genes responsible for flavonoid biosynthesis (CHS, DFR, ANS, and UFGT). The accumulated anthocyanins (AN) act as antioxidants to scavenge ROS and maintain cellular redox homeostasis. Anthocyanins can also decrease metal-induced cellular damage by chelating toxic metal ions and sequestering them into vacuoles. These defense systems together make the plant more resistant to adverse environmental conditions and maintain cellular integrity.

Reactive oxygen species (ROS) are not merely damaging byproducts of stress but important signaling molecules that regulate anthocyanin accumulation under adverse conditions [14]. Environmental stressors such as strong light, drought, and cold increase ROS generation, especially in chloroplasts. This in turn activates MYB–bHLH regulatory networks and anthocyanin biosynthesis genes such as *DFR* and *ANS* in *Arabidopsis* [15] (Figure 2).

ROS function both as stress signals and as components of feedback system in which anthocyanin accumulation contributes to oxidative buffering. Anthocyanin-deficient mutants typically exhibit elevated ROS levels and increased stress sensitivity, supporting the existence of a regulatory feedback loop between ROS homeostasis and pigment accumulation [15]. This dynamic system enables plants to maintain redox balance under fluctuating environmental stress. Comparative analyses across *Marchantia*, *Selaginella*, and *Arabidopsis* further suggest that anthocyanin-associated stress responses evolved alongside terrestrial adaptation through diversification of key biosynthetic genes such as *C4H* and *CHI2* [16]. Conservation of ROS-responsive anthocyanin regulation in angiosperms, lycophytes and bryophytes suggests that this protective stress-response mechanism originated early during land plant evolution and was further refined in higher plants. Furthermore, pigmentation responses linked to ROS can be amplified by species-specific regulators. For instance, in turnip (*Brassica rapa*), the ROS-promoting protein *BrLETM2* increases anthocyanin accumulation under stress [17]. These results corroborate the theory that ROS signaling plays a key upstream role in anthocyanin-driven dark phenotypes (ADPs), connecting coordinated pigment accumulation and oxidative buffering with stress perception. Therefore, understanding how ROS-responsive pathways regulate ADP production may open new possibilities for developing stress resistant crops with programmed anthocyanin responses.

### 3.2. Hormonal and Metabolic Integration in ADP Regulation

**Figure 3:**
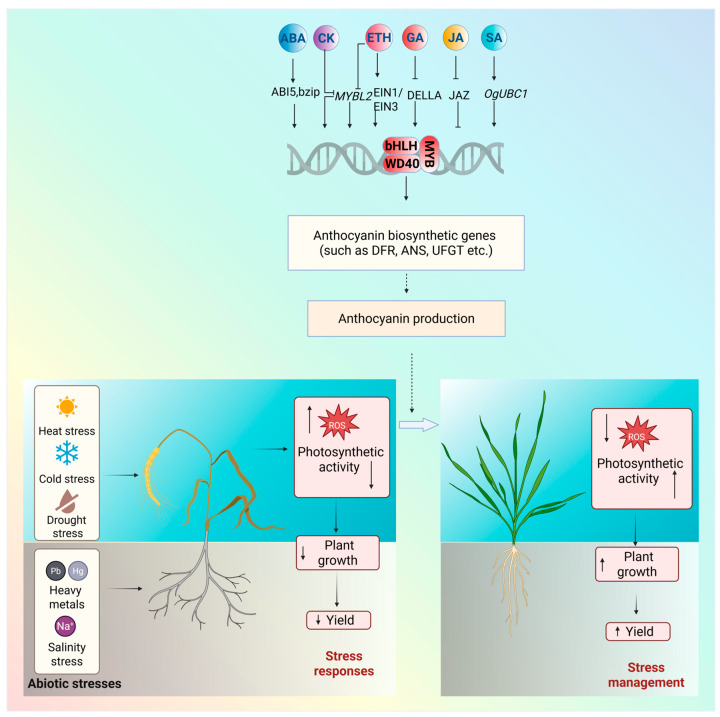
Schematic representation of phytohormone-mediated signaling networks coordinating anthocyanin biosynthesis and plant responses to abiotic stress. The phytohormone pathways that sense environmental stressors include abscisic acid (ABA), cytokinin (CK), ethylene (ETH), gibberellin (GA), jasmonic acid (JA) and salicylic acid (SA). These hormonal signals regulate some signaling elements and transcriptional regulators that converge on the MYB-bHLH-WD40 (MBW) transcriptional complex. The accumulation of anthocyanins is caused by the induction of the expression of genes involved in anthocyanin biosynthesis, such as DFR, ANS and UFGT, by the active MBW complex. Increased anthocyanin synthesis results in improved stress tolerance, ROS detoxification, protection of the photosynthetic machinery and maintenance of plant growth.

Anthocyanin-driven dark phenotypes (ADPs) are regulated through the integration of hormonal signaling and metabolic stress responses rather than through the action of a single pathway. Through common transcriptional regulators, including as ABFs, JAZ–MYC modules, and MYB–bHLH–WD40 (MBW) complexes, abscisic acid (ABA), jasmonic acid (JA), ethylene, and salicylic acid (SA) interact with sugar and energy signaling pathways to control anthocyanin production under stress (Figure 3) [18,19].

ABA signaling plays a central role in stress-induced pigmentation. By coordinating the activation of hormone signaling pathways, MYB-associated regulatory networks, and phenylpropanoid metabolism, exogenous ABA increases anthocyanin production in blueberry leaves [2]. The transcription factors MYBA1 and ABI5 serve as important positive regulators linking ABA signaling with anthocyanin biosynthetic gene expression [20]. ABI4, on the other hand, inhibits anthocyanin accumulation through HY5-dependent light signaling, suggesting that ABA-associated pathways balance environmental and developmental cues with stress-induced pigmentation [21]. Similarly, through H_2_O_2_-dependent ABA signaling, the *Arabidopsis abr* mutant accumulates higher anthocyanin levels and shows improved abiotic stress tolerance [22].

JA signaling play a important role in metabolic pathways, such as in *Panax ginseng*, the MeJA-responsive R2R3-MYB transcription factor PgMYB2 regulates the expression of a key ginsenoside biosynthetic gene [23]. JA signaling also plays a central role in coordinating stress-associated anthocyanin responses. High-light stress in rapeseed induces anthocyanin accumulation together with JA-responsive biosynthetic and regulatory genes, including *BnDFR*, *BnANS*, *BnPAP2*, and *BnGL3* [24]. By triggering ROS-scavenging pathways and anthocyanin-related genes, including IbCHI and IbDFR, the JA-responsive transcription factor IbMYC2 in sweet potatoes improves anthocyanin production and stress tolerance [25]. Furthermore, independent of classical JA signaling, the JAZ family member SmJAZ9 interacts with SmMYB113 to positively regulate anthocyanin formation in aubergine under high temperature and dark conditions [26]. These findings suggest that JA-associated regulatory modules integrate environmental stress signals with ADP formation.

Ethylene signaling exhibits context-dependent effects on anthocyanin accumulation. In certain systems, ethylene promotes light-induced anthocyanin biosynthesis through interactions with photosynthetic and malate metabolism pathways [27]. However, during fruit ripening, ethylene inhibits anthocyanin production in purple tomato by down-regulating the MYB regulator *SlAN2*-like and other biosynthetic genes [28]. This dual role indicates that ethylene integrates developmental and environmental signals to fine-tune anthocyanin accumulation.

SA signaling is also linked to anthocyanin-associated stress adaptation. Chilling stress induces SA accumulation and light-dependent anthocyanin biosynthesis in maize, suggesting coordinated regulation between phenylpropanoid metabolism and stress signaling pathways [29]. By lowering ROS formation, boosting antioxidant activity, and modifying stress responses linked to SA and ABA, anthocyanin therapy improves drought tolerance in rice [30].

Collectively, these studies indicate that rather than relying on distinct hormone-specific pathways, ADP control is dependent on broad hormonal crosstalk. While ethylene may either increase or decrease anthocyanin biosynthesis based on developmental and environmental context, ABA and JA typically work in concert to promote stress-responsive anthocyanin accumulation through ROS signaling, MBW-complex activation, and phenylpropanoid pathway induction. SA signaling strengthens antioxidant defense and stress adaptability by interacting with pathways linked to ROS and ABA. Plants can dynamically adjust anthocyanin accumulation and maximize stress tolerance under changing conditions due to the coordinated integration of hormonal, metabolic, and environmental cues.

All of these results lend credence to the theory that interrelated hormonal and metabolic signaling networks integrating transcriptional regulation, environmental signals, and cellular stress responses regulate ADP production. With programmable anthocyanin accumulation, these complex regulatory systems offer promising targets for stress-resilient crop engineering.

#### Sugar and Energy Sensing: The TOR–SnRK1 Metabolic Gate for ADP Formation

ADPs are tightly constrained by cellular energy status because anthocyanin biosynthesis is metabolically costly. Plants therefore couple pigment production to carbon and energy availability through the sucrose–TOR–SnRK1 signaling axis, where TOR (Target of Rapamycin) promotes growth under energy sufficiency, whereas SnRK1 (SNF1-Related Kinase 1) mediates energy conservation under deprivation [31]. TOR activity inhibits SnRK1 signaling in high sucrose environments, alleviating growth inhibition and enabling the activation of anthocyanin production pathways [32]. This promotes the transcriptional activation of genes involved in anthocyanin biosynthesis by stabilizing MBW-associated regulators such as MYB75/PAP1. On the other hand, SnRK1 becomes dominant and suppresses anthocyanin accumulation by targeting components of the MBW regulatory complex during low-energy or stress-related situations, which are frequently associated with lower “dark-phenotype” metabolic activity, thus limiting pigment formation [33]. In addition to controlling transcription, SnRK1 influences jasmonate signaling by phosphorylating and destabilizing JAZ repressors, such as JAZ18, which modifies bHLH activity and indirectly affects anthocyanin production. This establishes SnRK1 as a key mediator of pigment suppression, stress signaling, and energy restriction [32].

Crucially, the sucrose–TOR–SnRK1 axis serves as a metabolic gate for the production of ADP. While carbon constraint activates SnRK1, which puts survival programs ahead of secondary metabolism, elevated sugar levels activate TOR signaling and boost anthocyanin production [34]. This dual control ensures that anthocyanin accumulation and the associated dark coloration occur only when sufficient resources are available to support both biosynthesis and stress tolerance. Together, this energy-sensing network provides a mechanistic explanation for the conditional nature of anthocyanin-driven dark phenotypes, linking metabolic status with transcriptional regulation and environmental responsiveness.

## 4. MBW-Cantered Transcriptional Control of Anthocyanin-Driven Dark Phenotypes

ADPs are ultimately governed by transcriptional reprogramming centered on the MYB–bHLH–WD40 (MBW) complex, which represents the core regulatory module controlling late anthocyanin biosynthesis. Anthocyanins are widely distributed flavonoid pigments that support both ecological interactions, like pollinator attraction and stress tolerance. Monocots and dicots have different regulatory architectures for the conserved but adaptable MBW-dependent network that controls their biosynthesis [35,36].

In dicots like *Arabidopsis*, several R2R3-MYB, bHLH and WD40 proteins functionally interact in a combinatorial manner to control anthocyanin production, allowing for flexible control of tissue-specific and stress-responsive anthocyanin. Conversely, MYB and bHLH factors are more functionally specialized and developmentally coordinated in monocots, such as maize and barley, which depend more on lineage-specific regulatory modules [34]. In addition, monocots exhibit distinct regulatory hierarchies and spatial pigmentation patterns in both vegetative and reproductive tissues, indicating that MBW-dependent control has diversified during evolution, but that the activation of key genes involved in anthocyanin biosynthesis is conserved.

While late biosynthetic genes (LBGs) necessitate the formation of a functional MBW complex, early biosynthetic genes (EBGs) in dicots like *Arabidopsis* are primarily controlled by R2R3-MYB transcription factors [3,37]. Within this complex, the WD40 protein (TTG1) functions as a structural scaffold maintaining complex formation, bHLH proteins (such as GL3/TT8/EGL3) provide transcriptional activation capability via E-box binding, and R2R3-MYB proteins define target selectivity through promoter recognition [38,39]. This modular architecture ensures regulatory precision and environmental responsiveness.

By activating important biosynthetic genes, TTG1-like proteins (PaTTG1.1 and PaTTG1.2) in *Platanus acerifolia* increase anthocyanin accumulation, and their expression can compensate for *Arabidopsis ttg1* mutants [40]. Similarly, in barley, HvWD40-140 activates HvDFR by forming a functional MBW complex with HvANT1 and HvANT2, demonstrating conserved WD40-dependent regulation with species-specific modulation [41]. Together, these findings confirm that MBW complexes represent a conserved regulatory hub for anthocyanin biosynthesis across plant lineages.

**Figure 4:**
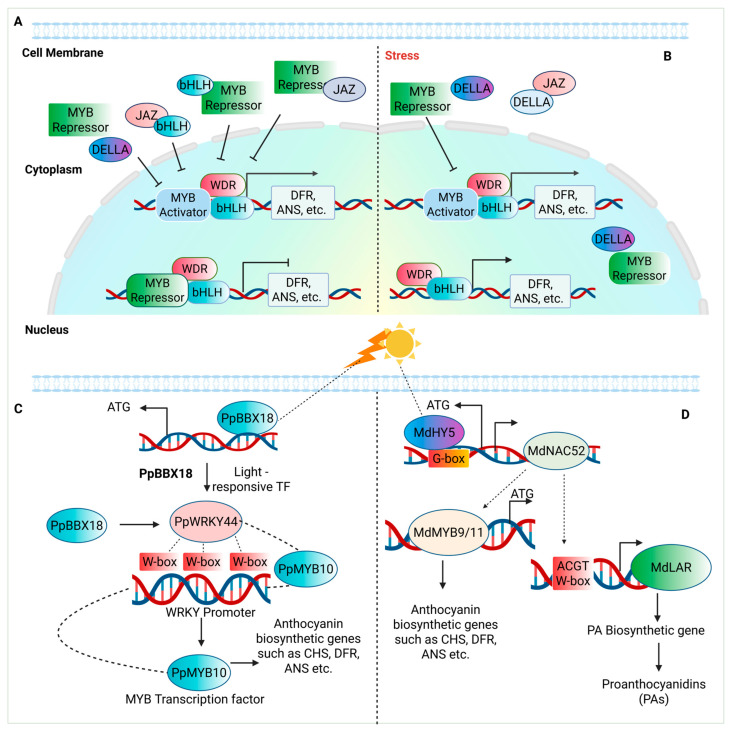
Integration of MBW core complex with WRKY and NAC transcriptional networks in anthocyanin-driven dark phenotypes. (**A**,**B**). The MBW complex acts as the central regulatory module controlling anthocyanin biosynthesis. Repressor proteins like JAZ and DELLA-associated complexes control the expression of genes involved in anthocyanin biosynthesis by inhibiting MBW activity under non-inductive circumstances. Stress signals release this repression, enabling the transcription of structural genes like CHS, DFR, and ANS as well as the activation of the MBW complex. (**C**). Together with WRKY proteins, light-responsive transcription factors like HY5 integrate environmental inputs and either directly or indirectly control anthocyanin biosynthesis genes and MBW components. (**D**). Upstream of the MBW complex, the NAC transcription factors directly regulate the genes encoding proteins involved in the biosynthesis of flavonoids or activate MYB genes associated with anthocyanins. Anthocyanin accumulation and photoprotection are linked to stress and developmental signals via WRKY and NAC factors.

Crucially, rather than operating independently, MBW activity is embedded within a multilayer regulatory network. Its activity is modulated by environmental and developmental cues like light, temperature, sugar status, phytohormones, and stress signals [42]. Furthermore, chromatin-associated regulators and small R3-MYB repressors fine-tune complex activity, ensuring context-dependent control of anthocyanin output. This multilayer regulation prevents excessive or maladaptive pigment accumulation while enabling rapid activation of anthocyanin production during stress. In the context of anthocyanin-driven dark phenotypes, the MBW complex serves as a central decision-making module that integrates upstream signals into downstream metabolic output (Figure 4A,B) [6]. Increased flux through the anthocyanin pathway results from sustained activation of late biosynthetic genes due to improved MBW stability and activator dominance. This increased flow gives ADPs their deep purple to black coloration and high optical density when combined with downstream mechanisms such as co-pigmentation, vacuolar sequestration, and pH modulation.

Thus, variation in MBW activity provides a mechanistic basis for the emergence of stress-induced dark phenotypes. This positions MBW regulation as a key engineering target for modulating anthocyanin accumulation and developing crops with enhanced stress resilience and photoprotective capacity.

### Integration of WRKY and NAC Networks into the MBW Regulatory Core

ADPs result from the MBW complex’s interaction with larger transcriptional networks, namely WRKY and NAC factors, even though it is the conserved core module governing anthocyanin biosynthesis. These regulators shape the intensity, duration, and spatial patterning of pigmentation by acting as both upstream modulators and downstream effectors of MBW activity [43] (Table 1). WRKY transcription factors serve as key regulatory hubs that connect MBW activity to environmental cues [44,45]. The MBW complex transcriptionally activates WRKYs in a number of systems, which then support anthocyanin biosynthesis through secondary regulatory programs, such as the activation of genes involved in late biosynthesis and the modification of vacuolar acidification and transport processes [43]. On the other hand, under specific stress or developmental conditions, some WRKYs act as context-dependent repressors that reduce MBW output, allowing for rapid adjustment of pigment accumulation. This dual regulatory activity explains their role in regulating both transient and sustained ADP states.

Direct integration between the WRKY and MBW modules is supported by experimental data. In grapevine, MYB5-associated MBW complexes recruit VvWRKY26 to jointly increase the expression of genes related to the flavonoid pathway [46]. By activating MYB regulators and biosynthetic genes in peaches and pears, light-responsive WRKYs like PpWRKY44 further link environmental cues to anthocyanin accumulation [47] (Figure 4C). WRKY factors, such as *Md*WRKY40, are involved in post-translational regulatory circuits in *Arabidopsis* and apple that control pigmentation amplitude and persistence, hence affecting the stability or transience of dark phenotypes [48].

**Table 1:** Transcription factors regulating anthocyanin accumulation in various crops under different stresses.

Stress Type	Transcription Factor	Crop	References
Abiotic stress	*bZIP*	*Morus alba*	[49]
Low Temperature	*R2R3-MYB*	*Cymbidium ensifolium*	[50]
Drought	*VvNAC17*	*Vitis vinifera*	[51]
Cold	*CBF*	*Oryza sativa*	[52]
Abiotic stress	*CstMYB1R1*	*Crocus sativus*	[53]
Abiotic	*bHLH*	*Glycyrrhiza uralensis*	[54]
High Light	*PuHB40*	*Pyrus ussuriensis*	[55]
Drought	*R2R3-MYB*	*Fagopyrum esculentum*	[56]
Cold	*OsLSC6*	*Oryza sativa*	[57]
Low temperature	*Ruby gene (MYB-like)*	*Citrus sinensis*	[58]
	*NtMYB4a*	*Nicotiana tabacum*	[59]
Cold, Heat, Drought and salinity	*HvANT1 HvANT2 HvWD40-140*	*Hordeum vulgare*	[60]
Ozone	*McWRKY71*	*Malus crabapple*	[61]
Abiotic	*LlMYB3*	*Lilium lancifolium*	[62]
Abiotic	*VcMYBL1; VcbHLHL1; VcWDL2*	*Vaccinium corymbosum*	[2]
Abiotic	*DREB*	*Ammopiptanthus mongolicus*	[63]
High Sucrose and Oxidative and Abiotic Stresses	*ANAC032*	*Arabidopsis*	[64]
UV radiation, Temprature	*VvMYBA1, VvMYBA2 VvMYC1 VvWDR1*	*Vitis vinifera*	[65]

NAC similarly integrates into the MBW-centered regulatory network, acting as both repressors and activators depending on context (Figure 4D). By downregulating TT8 and late biosynthetic genes like *DFR* and *ANS*/*LDOX* in *Arabidopsis*, ANAC032 inhibits anthocyanin accumulation while concurrently boosting endogenous repressors like MYBL2 and SPL9, which attenuates MBW activity under various stress situations [64]. On the other hand, *Md*NAC77L directly activates the promoters of important biosynthetic genes (*DFR*, *ANS*, *UFGT*) to increase anthocyanin accumulation in apples and strawberries [66]. Similarly to this, MdNAC52 incorporates light signals into the regulation of flavonoids by coordinating the manufacture of anthocyanins and proanthocyanidins, activating MYB9/MYB11, and controlling structural genes like LAR [67].

When WRKY and NAC transcription factors function together, the MBW regulatory core becomes a multilayered transcriptional network that incorporates metabolic, hormonal, and environmental inputs. The creation, reversibility, and stability of anthocyanin-driven dark phenotypes (ADPs) are shaped by the precise control over anthocyanin accumulation made possible by this hierarchical integration (Figure 4C,D; Table 1).

## 5. Epigenetic Control of Anthocyanin-Driven Dark Phenotypes

Depending on the epigenetic status of anthocyanin regulatory networks, ADPs can either remain transient or persist through cell divisions. Epigenetic mechanisms offer a higher-order regulatory layer that governs the accessibility of anthocyanin biosynthesis and regulatory genes, going beyond transcription factors and signaling pathways. The stability, strength, and heredity of pigmentation responses are influenced by DNA methylation, histone modifications and chromatin remodeling, which together decide whether important loci remain transcriptionally active or repressed [68,69]. By means of these pathways, plants are able to create transcriptional memory of past environmental stressors, which allows for either quick reversal once stress conditions are removed or chronic anthocyanin buildup. Therefore, epigenetic remodeling acts as a biological link between long-term phenotypic outcomes and environmental perception [70,71].

**Figure 5:**
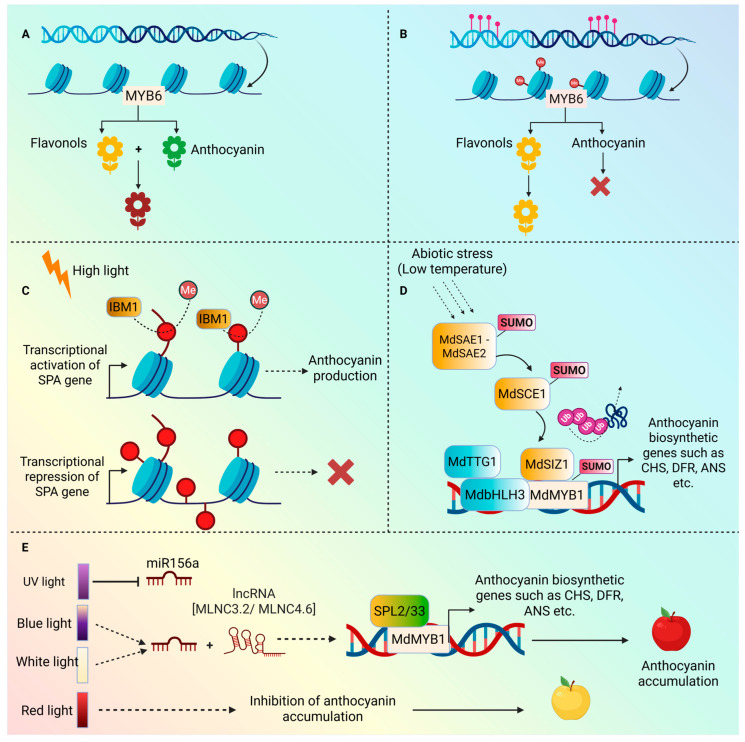
Multilayered transcriptional, epigenetic, post-translational, and light-responsive regulation of anthocyanin biosynthesis under developmental and environmental cues. (**A**,**B**). Through changes in transcription factor binding and gene expression, chromatin accessibility and epigenetic alterations control genes involved in anthocyanin biosynthesis. Through alterations in chromatin structure, DNA methylation and histone modifications can either encourage or inhibit anthocyanin accumulation (**C**). High light modifies *IBM1*-associated histone methylation, which activates SPA genes transcriptionally and increases anthocyanin synthesis. Repressive chromatin marks, on the other hand, prevent pigment production and suppress SPA expression. (**D**). Low temperatures trigger SUMOylation pathways involving *MdSAE1/2*, *MdSCE1*, and *MdSIZ1*. As a result, genes involved in anthocyanin production are highly activated by MBW components (*MdMYB1*–*MdbHLH3*–*MdTTG1*) that have been modified by SUMO. (**E**). To regulate anthocyanin formation and fruit coloration, light-quality signaling pathways combine red, blue, and UV light cues via photoreceptors, transcription factors, and regulatory RNAs. The intensity, stability, and environmental responsiveness of anthocyanin synthesis are all coordinated by these regulatory levels.

Chromatin-level mechanisms serve as a higher-order regulatory layer that controls the persistence, stability, and memory of the dark phenotypic state, in contrast to ROS, hormonal, and metabolic signals that primarily influence the production of ADPs. In this context, chromatin accessibility—including DNA methylation and histone modifications that determine whether anthocyanin biosynthesis and regulatory loci are transcriptionally competent—regulates MBW transcriptional activity, which is not autonomous (Supplementary Table S1) [72,73]. The *chrysanthemum* study demonstrates that while reduced methylation restores *CmMYB6* expression and pink pigmentation (YP-P), stable CHH methylation of the *CmMYB6* promoter silences this important anthocyanin-activating MYB, resulting in yellow flowers (YP-Y) [74] (Figure 5A,B). Likewise, bright light causes *Arabidopsis* to produce the histone demethylase *IBM1*, which eliminates H3K9me2 at SPA loci, modifying COP1–SPA repression and indirectly stabilizing MBW components [72] (Figure 5C). Additionally, genome-wide methylome reprogramming during light-induced pear coloration demonstrates that structural genes (*PyUFGT*, *PyDFR*, and *PyANS*) are activated by CHH hypomethylation without changing the MYB10 coding sequence [75]. Unlike the mutation-driven promoter methylation observed in *chrysanthemum*, this environmentally mediated epigenetic reprogramming increases transcription and pigment accumulation without changing MYB10.

Together, these studies reveal two common epigenetic mechanisms of ADPs: (i) DNA methylation-based regulation of structural genes and anthocyanin regulators, and (ii) histone modification-based alterations in chromatin accessibility. These processes act as molecular switches that determine whether stress-induced pigmentation becomes mitotically stable or transient. Chromatin remodeling also contributes to stress memory and persistence of dark phenotypes by generating epigenetic memory, which can maintain transcriptional competence of anthocyanin pathways even after the initial stimulus fades. Therefore, epigenetic regulation provides promising opportunities for breeding crops with long-lasting anthocyanin-mediated stress tolerance and constitutes a key interference for the translation of environmental cues into heritable pigmentation states.

### 5.1. Post-Translational Control

Even in situations when transcription is permissive, protein-level control is essential for anthocyanin activation. The stability, activity, and interaction capacity for interaction of MYB, bHLH, and WD40 factors, as well as their partner regulators, are greatly impacted by phosphorylation, ubiquitination, SUMOylation, and targeted proteolysis [76]. As demonstrated by MPK4–MYB75 in *Arabidopsis* and MdMPK6–MdHY5 in apples, MAPK-dependent phosphorylation stabilizes important transcription factors under light, promoting the accumulation of anthocyanins [77,78]. ROS-dependent phosphorylation in pear similarly reinforces MBW activation under high-light stress [55]. In addition, *MdSIZ1*-mediated SUMOylation of *MdMYB1* in red-skinned apples promotes anthocyanin accumulation and red fruit coloration by stabilizing this MYB under low temperature and phosphorus deprivation [79] (Figure 5D).

Unlike the light-triggered MAPK pathways in *Arabidopsis* (MPK4–MYB75) and apple (MdMPK6–MdHY5), this mechanism exhibits a stress-responsive, ROS-dependent phosphorylation route that converges on MBW-like transcriptional complexes. All these studies indicate that post-translational modifications, particularly phosphorylation, act as flexible regulatory nodes that integrate environmental light cues and oxidative stress signals to precisely control transcription factor activity and anthocyanin accumulation across species. This demonstrates how plants can coordinate pigmentation with various environmental inputs using conserved molecular processes. Both studies agree that post-translational changes in response to environmental cues dynamically alter the stability of MBW transcription factors, adding a crucial layer of regulatory flexibility beyond transcription and chromatin state. These findings suggest that SUMOylation and phosphorylation function as reversible molecular switches, adjusting MBW activity to integrate stress and light cues into anthocyanin biosynthesis. In contrast, degradation mediated by ubiquitin provides a balancing mechanism. Proteins such as MdBT2 and FvCSN5 facilitate the breakdown of MBW regulators and prevent excessive pigment accumulation [80,81]. Furthermore, nutrient-responsive modules (such as MdBT2-MdGRF11 and MdPHR1-MdSINA1) demonstrate how ubiquitination functions as a dynamic rheostat that links pigment synthesis with nutritional status [82].

Post-translational control allows for the quick and reversible deployment of dark pigmentation by dynamically adjusting MBW stability and activity. This provides a versatile molecular framework for creating stress-resilient crops without permanently changing growth programs.

#### Non-Coding RNAs in Anthocyanin Control

Although transcription factors and chromatin regulators define much of the anthocyanin output, an extra regulatory tier is imposed by small and long non-coding RNAs (miRNAs, siRNAs, and lncRNAs) that operate as post-transcriptional gatekeepers of pigment production. These RNA species enable quick changes in pigmentation in response to light, nutritional cues, and stress by fine-tuning the quantity, translation, or epigenetic state of anthocyanin-related genes [83]. Interestingly, a number of recent studies demonstrate that ncRNAs can function as competing endogenous RNAs to buffer MBW-related transcription factors, activate structural genes via siRNA-directed demethylation, or modify MYB repressors [84]. Because dark anthocyanin phenotypes often result from increased stability or derepression of positive regulators, ncRNA circuits are a crucial mechanism that allows plants to maintain high pigment levels even in low-light or poor environments. Thus, miRNAs, siRNAs, and lncRNAs form a new regulatory layer that combines persistent or stress adaptive accumulation of anthocyanin with environmental sensing.

During development and in response to light, miRNA modules like miR156–SPL and miR858–MYB regulate MBW activity, whilst lncRNAs can function as hormone-linked amplifiers or endogenous target mimics [85,86] (Figure 5E). Spatial patterning studies in dahlia and systems-level analyses in poplar further demonstrate that ncRNAs reorganize transcriptional hierarchies controlling flavonoid metabolism [87]. Nevertheless, the majority of the available data points to ncRNAs as modulators of inducible pigmentation rather than the main cause of persistently dark phenotypes. Chromatin remodeling or long-term stability of MBW proteins are more frequently linked to constitutive or persistent dark coloration. One significant unexplored area is integrative profiling, which combines chromatin status, TF stability, and ncRNA dynamics in low-light conditions. Anthocyanin regulation is made more precise and spatially specific by non-coding RNA circuits, which implies that targeted RNA-based techniques could optimize dark phenotypes to improve stress adaptation while reducing growth and energy allocation cost.

## 6. Engineering Anthocyanin-Driven Dark Phenotypes for Climate-Resilient Crops Systems

**Figure 6:**
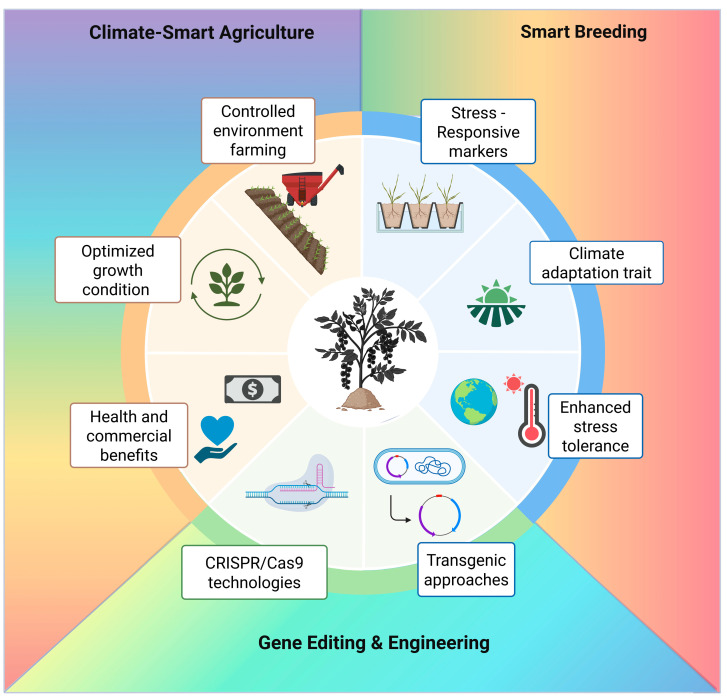
An integrated framework for improving climate-resilient crops that connects gene editing, smart breeding, and climate-smart agriculture. By using controlled surroundings, optimizing growing conditions, and offering both health and financial benefits, climate-smart agriculture increases crop yields. To create resilient varieties, clever breeding methods make use of climate-adaptive characteristics and stress-responsive markers. Additionally, the development of crops with enhanced stress tolerance and yield stability is accelerated by gene editing and engineering techniques like CRISPR/Cas9 and transgenic technologies.

Anthocyanin-driven dark phenotypes (ADPs) represent coordinated, stress-adaptive regulatory states shaped by chromatin remodeling, transcriptional control, hormonal signaling, and metabolic reprogramming. ADPs function as integrative physiological configurations that reflect the activation of endogenous stress-protective networks, not merely as an extension of pigmentation intensity but as a system-level response. By treating dark pigmentation as a programmable feature, this conceptual shift links translational crop engineering techniques with a mechanistic understanding of stress signaling. According to this theory, ADPs are visible outcomes of integrated regulatory systems that include metabolic gating, ROS signaling, MBW-centered transcriptional control, and epigenetic regulation. Consequently, dark pigmentation becomes a functional engineering target for enhancing crop resilience under climate variability, rather than just a descriptive attribute (Figure 6).

### 6.1. Molecular Engineering of ADPs via Regulatory and Pathway Control

Direct proof that ADPs can be created by specifically altering biosynthetic and regulatory genes is provided by anthocyanin regulatory module alteration. By triggering stress-responsive metabolic networks, overexpression of miR393 in *Brassica napus* increases anthocyanin accumulation and strengthens resistance to oxidative and osmotic stress [88]. Under cold and freezing stress, anthocyanin biosynthesis genes like ANS, which are controlled by BrMYB2-2 and BrTT8, exhibit substantial induction in *Brassica rapa*, connecting pigment accumulation with stress adaptation [89]. Similarly to this, DFR overexpression in B. napus increases anthocyanin levels, decreases ROS formation, and increases resistance to osmotic stress and salinity, indicating that several enzymatic nodes in the flavonoid pathway contribute to stress resilience [90]. The engineering potential of ADPs is further reinforced by cross-species regulation conservation. By increasing antioxidant and detoxifying pathways, RsMYB1 overexpression in petunia increases anthocyanin formation and strengthens resistance to heavy metal stress (Ai et al. 2018) [91]. In parallel, melatonin-induced stress tolerance under chromium toxicity is mediated through anthocyanin biosynthesis, where suppression of ANS abolishes protection, demonstrating that anthocyanins act as essential downstream effectors in stress mitigation (Sun et al. 2023) [92]. Taken together, these investigations show that anthocyanin accumulation is both a functional characteristic and a mechanistic indicator of stress resilience, establishing a clear connection between pigment modulation and adaptive performance.

ADPs provide an integrative and visible proxy for stress-responsive regulatory network activation in modern breeding systems. Dark pigmentation is a strong phenotypic indicator of stress competence because, unlike single-gene tolerance markers, it reflects the coordinated output of ROS signaling, hormonal integration, MBW activity, and metabolic flux regulation. Along with increased antioxidant enzyme activity, improved ion homeostasis, reduced oxidative damage, and upregulation of stress-responsive transcription factors like *SlDREB2A*, *SlWRKY8*, *SlABF4*, and *SlAREB1*, the anthocyanin-rich tomato genotype LA-1996 demonstrates superior tolerance to salinity and drought stress [93]. Principal component analysis further confirms anthocyanin accumulation as a key trait associated with multi-stress resilience in this genotype. Anthocyanin-based visual markers, such as the R1-Navajo system, enable quick haploid identification and effective selection in maize doubled haploid (DH) systems. However, its performance in tropical germplasm is limited by C1-I allele inhibition, which has been addressed through molecular marker-based prediction of inhibitory alleles (Chaikam et al. 2015) [94]. This demonstrates how regulatory insight into anthocyanin pathways can directly increase the accuracy and productivity of breeding [94]. The value of anthocyanins as selection traits across crops is supported by additional data. In recurrent selection systems, artificial anthocyanin buildup in rice acts as a reliable visual indicator [95]. Stress-responsive anthocyanin accumulation in purple wheat, however, is correlated with yield formation and developmental stage, underscoring its dual physiological and agronomic significance [96]. When taken as a whole, this research establishes ADPs as useful indicators for incorporating stress biology into breeding processes.

All these studies indicate a single principle underlying ADPs: increased anthocyanin accumulation consistently correlates with the activation of antioxidant defenses, maintenance of ROS homeostasis, and induction of stress-responsive regulatory networks, regardless of the upstream regulatory factor, genetic modification strategy, or stress condition. Therefore, ADPs are not merely a pigmentation feature, but an integrated and observable product of coordinated stress-adaptation systems. This association of dark pigmentation with stress resilience supports the use of ADPs as a valuable phenotypic marker for multi-stress resistance breeding. This enables rapid screening for resilient genotypes and reduces the need for complex physiological or molecular assays.

### 6.2. From Molecular Regulation to Climate—Smart Deployment ADPs

**Figure 7:**
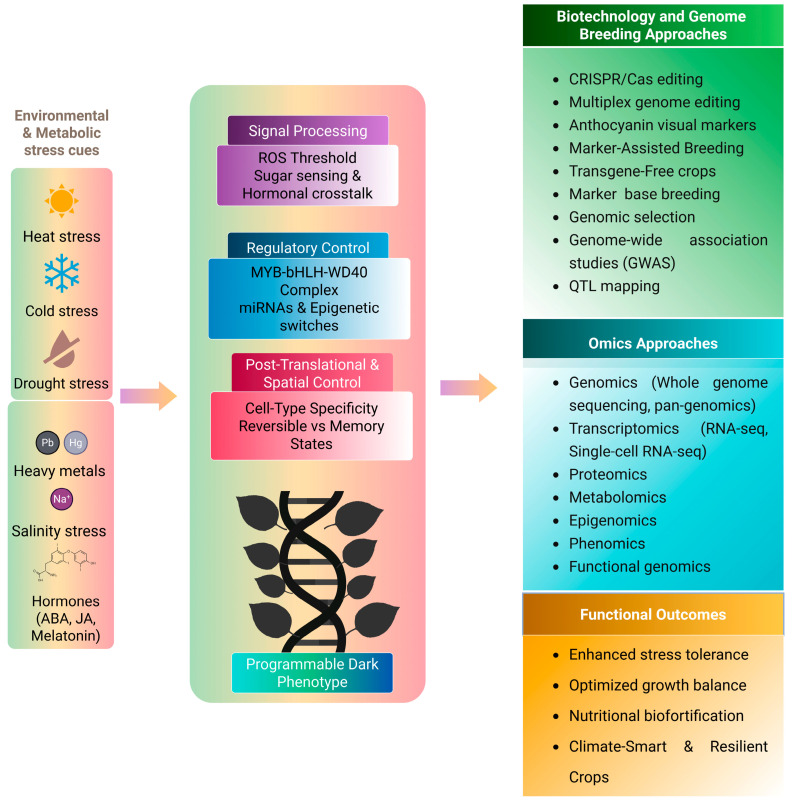
Systems-level framework for engineering stress-responsive dark phenotypes to enhance climate resilience in crops. Reactive oxygen species (ROS), hormonal signaling pathways, and regulatory networks centered on MYB–bHLH–WD40 proteins are involved in processing environmental and metabolic stress signals, leading to the development of programmable dark phenotypes. These insights are used to create climate-smart, nutritionally improved, and stress-resilient crops, using omics, genome breeding, and gene-editing techniques.

Only when the underlying regulatory logic of anthocyanin-driven dark phenotypes (ADPs) is expanded beyond molecular control to include crop design and agricultural deployment will they achieve translational usefulness. ADPs integrate ROS signaling, hormonal crosstalk, MBW-centered transcriptional control, and epigenetic modification into a single, selectable trait, serving as observable system-level readouts of coordinated stress-response networks. This systems perspective enables mechanistic insights to be transformed into implementable solutions for climate-resilient agriculture (Figure 7). A tiered pipeline linking environmental control, breeding strategies, and gene-level modification can operationalize ADPs at the engineering scale.

While omics-guided network reconstruction enables the identification of stable regulatory hubs controlling pigment intensity and stress responsiveness, gene-editing and transgenic techniques (such as CRISPR/Cas-mediated regulatory rewiring of anthocyanin repressors or activators) allow for precise tuning of pathway flux [97,98]. Predictive phenotyping platforms that measure anthocyanin accumulation as a stand-in for stress resistance complement these genetic interventions, allowing for the early selection of resilient genotypes in a variety of environmental conditions.

In a recent study genome-wide investigation of R2R3-MYB transcription factors revealed that *OsMYB1* acts as a key negative regulator of anthocyanin production in rice [99]. *OsMYB1* inhibits structural genes (*OsDFR*, *OsANS*, and *OsF3′H*) and engages with the MBW complex to regulate pigmentation. The CRISPR-Cas9 deletion of *OsMYB1* in purple rice types resulted in increased anthocyanin accumulation in leaves and pericarps, thereby upregulating biosynthetic genes. While in the purple tomato cultivar ‘Indigo Rose’, CRISPR/Cas9 was used to mutate the R2R3-MYB transcription factor *SlAN2*, demonstrating its regulatory role in anthocyanin accumulation [100]. This targeted mutagenesis of *SlAN2* specifically reduced anthocyanin levels in vegetative tissues, without affecting fruit pigmentation, highlighting tissue-specific control of dark phenotypes. When taken together, the two studies show how CRISPR-based modifications of key regulatory genes can be used to precisely control dark phenotypes. This enhances our ability to link pigmentation to nutritional value, developmental traits, and stress resilience. These techniques provide a solid basis for improving climate-resilient crops, allowing the use of phenotypes high in anthocyanins to support biofortification and robustness in a range of crops. In black and white rice, the transcriptional activator *OsC1* further exemplifies the dual utility of anthocyanins in plant stress physiology [101]. *OsC1* overexpression improved oxidative stress tolerance, increased photosynthetic efficiency, and reduced membrane damage by upregulating late biosynthesis genes, which in turn caused anthocyanin accumulation. Dynamic physiological characteristics of ADP states include reversible activation under changing environmental conditions, metabolic trade-offs between growth and secondary metabolism, and possible involvement in stress-associated memory. These characteristics demonstrate that ADPs are flexible regulatory states that balance stress protection and resource allocation rather than representing static pigmentation outcomes. However, developmental stage, stress level, and environmental history all affect the size and stability of these reactions, indicating that threshold-dependent regulation controls ADP activation and deactivation.

Importantly, effective agronomic induction techniques that operate upstream of genetic or breeding interventions are also necessary for the deployment of ADP in climate-smart agriculture. Anthocyanin-based stress responses can be activated through a scalable entry point provided by environmental preconditioning during early embryonic stages. Through photoreceptor-mediated activation of MBW regulatory networks, manipulation of light, such as increased blue and red-to-far-red ratios or brief UV-B exposure, can enhance anthocyanin production. Similarly, ROS-mediated signaling that primes defensive pigmentation pathways is triggered by temperature priming (moderate cold or heat treatments) during seedling establishment. Without genetic modification, nutrient adjustment, specifically nitrogen restriction or phosphate modulation, interacts with hormone signaling to precisely regulate ADP expression. By enabling reversible, stage-specific activation of dark phenotypes, these environmentally generated cues connect molecular regulation with field-level agronomic application in climatically changeable environments.

Crucially, ADP-based engineering includes integrated agronomic management as well as genetic modification. Without permanently altering the DNA, endogenous anthocyanin regulatory networks can be activated through environmental tuning techniques such as temperature priming, regulated light quality, and nutrition manipulation. As a result, stress-adaptive pigmentation becomes both inducible and reversible, depending on production requirements, creating a variable interface between genotype and environment.

These diverse approaches combine to create a cohesive climate-smart framework that integrates environmental control, breeding, and molecular engineering. In this concept, ADPs connect intracellular regulatory logic with field-level performance results by acting as quantifiable outputs of stress-responsive network activation. By identifying ROS signaling, hormonal networks, and MBW-centered transcriptional regulation as the primary integrators connecting environmental stress perception to programmable pigmentation states, Figure 7 further synthesizes this idea. Together, these systems-level viewpoints reframe anthocyanin-based dark phenotypes as designed, scalable, and environmentally responsive modules for climate-resilient crop design rather than as isolated metabolic features.

## 7. Conclusions

Rather than being passive metabolic outputs, anthocyanin-driven dark phenotypes (ADPs) are better characterized as integrated stress-adaptive states. The coordinated regulation of ROS signaling, hormonal crosstalk, MBW-centered transcriptional control, and epigenetic modulation is reflected in dark pigmentation across plant systems, which connects increased physiological resistance to environmental stress perception. These regulatory networks can now be precisely tuned thanks to developments in CRISPR-based editing and multi-omics techniques, making ADPs programmable features for climate-resilient crop engineering. The metabolic balance between growth and anthocyanin accumulation, determining the exact stress thresholds that cause advantageous pigmentation responses, and guaranteeing the phenotypic stability of ADPs across developmental stages and changing environments are some of the major issues that are still unsolved. For their dependable use in crop systems, these gaps must be filled. In order to address cell-type-specific regulation of ADPs, future research should integrate single-cell and spatial omics technologies with field-level validation under dynamic environmental settings to evaluate ecological performance and stability. In order to translate ADPs from mechanistic insights into stable, predictable, and deployable traits for crop development under climate variability, such integrated techniques will be essential.

## Data Availability

No new data were created or analyzed in this study.

## Supplementary Materials

The following supporting information can be downloaded at: https://www.mdpi.com/article/10.3390/plants15121870/s1. References [102,103,104,105,106] are cited in the supplementary materials.
