# Supplementary material for: Anthocyanin-Driven Dark Phenotypes in Stress Adaptation
Source: Plants (Basel). 2026 Jun 16;15(12):1870. doi: 10.3390/plants15121870 (PMC13306268; doi:10.3390/plants15121870)
Supplement: Supplementary file 1 [file plants-15-01870-s001.zip › plants-4343055-supplementary.pdf]

Supplementary Table S1: Multilayer regulatory network controlling anthocyanin-driven dark phenotypes across plant species.

Regulatory Layer	Key Component	Target Gene/Protein	Mechanism	Environmental Trigger	Species	Functional Outcome	Reference
DNA methylation (CHH)	Promoter methylation of *PrANS*/*PrF3H*; *R2R3*-MYBs (*PrMYBa1*/*PrMYBa3*) + bHLHs	*PrF3H, PrDFR, PrANS*	Promoter hypermethylation MYB–bHLH activation enable blotch-specific expression	Developmental regulation	*Paeonia rockii 'Shu Sheng Peng Mo'*	Localized anthocyanin accumulation forming petal blotches	[1]
DNA methylation	Promoter methylation of *MdMYB10*; MBW complex (MdMYB10–MdbHLH3–MdWD40)	*MdMYB10* and downstream anthocyanin biosynthetic genes	Higher promoter methylation of *MdMYB10* in striped-skin apples reduces TF expression	Developmental/light cue	*Malus domestica*	Differential anthocyanin accumulation resulting in blushed vs. striped skin coloration	[2]
H3K9 demethylation & non-CG DNA	*IBM1* (JmjC histone demethylase) regulating	*SPA1/3/4* (*COP1/SPA* complex components)	IBM1 demethylates H3K9 and non-CG DNA at SPA loci,	High light (HL) stress	*Arabidopsis thaliana*	Modulation of light-induced anthocyanin accumulation	[3]

hypomethylation	SPA1/3/4 chromatin		activating their expression under HL				
Chromatin remodeling–mediated epigenetic regulation (H2A.Z deposition vs. H3K4me3 modification)	H2A.Z (SWR1 complex) antagonistic to H3K4me3	Anthocyanin biosynthetic genes	H2A.Z deposition represses target genes, while reduced H2A.Z increases H3K4me3 and activates transcription	Developmental/ environmental cues	*Arabidopsis thaliana*	Epigenetic control of anthocyanin accumulation	[4]
Post-translational regulation (MAPK-mediated phosphorylation)	MPK4 phosphorylating MYB75/PAP1	MYB75/PAP1 (anthocyanin regulator)	MPK4 phosphorylates and stabilizes MYB75, enhancing its transcriptional activity	Light	*Arabidopsis thaliana*	Light-induced anthocyanin accumulation	[5]
Light-responsive	MdHY5 (bZIP TF)	MdMYB10 and	MdHY5 binds G-box/E-box motifs to	Light (and ABA)	*Malus domestica*	Enhanced anthocyanin	[6]

transcriptional regulation (bZIP-mediated)		anthocyanin biosynthetic genes	activate MdMYB10 and downstream genes			accumulation (and nitrate assimilation)	
Post-translational regulation (SUMOylation-mediated stabilization)	MdSIZ1 (SUMO E3 ligase)	MdMYB1 (MBW complex regulator)	MdSIZ1 SUMOylates MdMYB1, enhancing its stability and transcriptional activity	Moderately low temperature, phosphorus deficiency	*Malus domestica*	Increased anthocyanin accumulation and red fruit coloration under stress	[7]
Post-translational regulation (ubiquitin-mediated degradation)	MdBT2 (BTB-TAZ protein)	MdMYB1 (anthocyanin regulator)	MdBT2 promotes ubiquitination and proteasomal degradation of MdMYB1	Nitrate	*Malus domestica*	Suppression of anthocyanin accumulation and fruit coloration	[8]
Post-translational regulation (ubiquitin–	*FvCSN5* (COP9 signalosome subunit)	*FvMYB1* and *FvBBX20*	*FvCSN5* promotes ubiquitin-mediated degradation of MYB1 and BBX20, modulating	Not specified	*Fragaria vesca* (woodland strawberry)	Reduced anthocyanin accumulation in fruit	[9]

proteasome pathway)			downstream gene expression				
Post-transcriptional regulation (miRNA-mediated SPL control)	*miR156–SPL9 module*	MBW complex and anthocyanin biosynthetic genes	SPL9 reduces anthocyanin genes by destabilizing the MYB–bHLH–WD40 complex; miR156 inhibits SPL9 to encourage anthocyanin accumulation.	Developmental phase transition (vegetative to flowering)	*Arabidopsis thaliana*	Developmentally controlled equilibrium between the accumulation of flavonols and anthocyanins	[10]
Post-transcriptional regulation (lncRNA-mediated activation of hormone biosynthesis)	*MdLNC610 (lncRNA)*	*MdACO1* (ethylene biosynthesis gene)	*MdLNC610* makes *MdACO1* work harder, which makes more ethylene and starts the process of making anthocyanins.	High light	*Malus domestica*	Better fruit coloring and anthocyanin buildup brought on by ethylene	[11]

Reference

1. Zhu, J.; Wang, Y.; Wang, Q.; Li, B.; Wang, X.; Zhou, X.; Zhang, H.; Xu, W.; Li, S.; Wang, L. The Combination of DNA Methylation and Positive Regulation of Anthocyanin Biosynthesis by MYB and bHLH Transcription Factors Contributes to the Petal Blotch Formation in Xibei Tree Peony. *Horticulture research* **2023**, *10*, uhad100.
2. Jia, D.; Li, Z.; Dang, Q.; Shang, L.; Shen, J.; Leng, X.; Wang, Y.; Yuan, Y. Anthocyanin Biosynthesis and Methylation of the *MdMYB10* Promoter Are Associated with the Red Blushed-Skin Mutant in the Red Striped-Skin "Changfu 2" Apple. *J. Agric. Food Chem.* **2020**, *68*, 4292–4304, doi:10.1021/acs.jafc.9b07098.
3. Fan, D.; Wang, X.; Liu, T.; Liu, H.; Peng, Y.; Tang, X.; Ye, X.; Sun, K.; Yue, Y.; Xu, D.; et al. Epigenetic Regulation of High Light-induced Anthocyanin Biosynthesis by Histone Demethylase IBM1 in *Arabidopsis*. *New Phytologist* **2024**, *242*, 2570–2585, doi:10.1111/nph.19789.
4. Cai, H.; Zhang, M.; Chai, M.; He, Q.; Huang, X.; Zhao, L.; Qin, Y. Epigenetic Regulation of Anthocyanin Biosynthesis by an Antagonistic Interaction between H2A.Z and H3K4me3. *New Phytologist* **2019**, *221*, 295–308, doi:10.1111/nph.15306.
5. Li, S.; Wang, W.; Gao, J.; Yin, K.; Wang, R.; Wang, C.; Petersen, M.; Mundy, J.; Qiu, J.-L. MYB75 Phosphorylation by MPK4 Is Required for Light-Induced Anthocyanin Accumulation in Arabidopsis. *The Plant Cell* **2016**, *28*, 2866–2883.
6. An, J.-P.; Qu, F.-J.; Yao, J.-F.; Wang, X.-N.; You, C.-X.; Wang, X.-F.; Hao, Y.-J. The bZIP Transcription Factor MdHY5 Regulates Anthocyanin Accumulation and Nitrate Assimilation in Apple. *Horticulture research* **2017**, *4*.
7. Zhou, L.; Li, Y.; Zhang, R.; Zhang, C.; Xie, X.; Zhao, C.; Hao, Y. The Small Ubiquitin-like Modifier E3 Ligase MdSIZ1 Promotes Anthocyanin Accumulation by Sumoylating MdMYB1 under Low-temperature Conditions in Apple. *Plant Cell & Environment* **2017**, *40*, 2068–2080, doi:10.1111/pce.12978.
8. Wang, X.-F.; An, J.-P.; Liu, X.; Su, L.; You, C.-X.; Hao, Y.-J. The Nitrate-Responsive Protein MdBT2 Regulates Anthocyanin Biosynthesis by Interacting with the MdMYB1 Transcription Factor. *Plant Physiology* **2018**, *178*, 890–906.
9. Nie, Y.; Lei, Y.; Jiao, H.; Zhang, Z.; Yao, J.; Li, H.; Dai, H.; Zhang, Z.; Zhang, J. Ubiquitin-mediated Degradation of the Inhibitor FvMYB1 and the Activator FvBBX20 by FvCSN5 Balances Anthocyanin Biosynthesis in Strawberry Fruit. *The Plant Journal* **2025**, *121*, e70021, doi:10.1111/tpj.70021.
10. Gou, J.-Y.; Felippes, F.F.; Liu, C.-J.; Weigel, D.; Wang, J.-W. Negative Regulation of Anthocyanin Biosynthesis in Arabidopsis by a miR156-Targeted SPL Transcription Factor. *The Plant Cell* **2011**, *23*, 1512–1522.
11. Yu, J.; Qiu, K.; Sun, W.; Yang, T.; Wu, T.; Song, T.; Zhang, J.; Yao, Y.; Tian, J. A Long Noncoding RNA Functions in High-Light-Induced Anthocyanin Accumulation in Apple by Activating Ethylene Synthesis. *Plant Physiology* **2022**, *189*, 66–83.
